# Supplementary material for: Comparative genomics provides new insights into the diversity, physiology, and sexuality of the only industrially exploited tremellomycete: Phaffia rhodozyma
Source: BMC Genomics. 2016 Nov 9;17:901. doi: 10.1186/s12864-016-3244-7 (PMC5103461; doi:10.1186/s12864-016-3244-7)
Supplement: Additional file 6: — List of orphan genes with links to PFAM (related to Additional file 1: Table S1). (ZIP 1428 kb) [file 12864_2016_3244_MOESM6_ESM.zip › BLAST_HTML_FTR/G02131_P.html]

BLAST Search Results


```
BLASTP 2.2.27+


Reference:
Stephen F. Altschul, Thomas L. Madden, Alejandro A. Schäffer,
Jinghui Zhang, Zheng Zhang, Webb Miller, and David J. Lipman (1997),
"Gapped BLAST and PSI-BLAST: a new generation of protein database
search programs", Nucleic Acids Res. 25:3389-3402.


Reference for
composition-based statistics:
Alejandro A. Schäffer, L. Aravind, Thomas L. Madden, Sergei
Shavirin, John L. Spouge, Yuri I. Wolf, Eugene V. Koonin, and
Stephen F. Altschul (2001), "Improving the accuracy of PSI-BLAST
protein database searches with composition-based statistics and
other refinements", Nucleic Acids Res. 29:2994-3005.


Database: nr
           71,551,133 sequences; 26,053,659,533 total letters


Query= G02131_P

Length=143
                                                                      Score     E
Sequences producing significant alignments:                          (Bits)  Value

emb|CED82143.1|  hypothetical protein [Xanthophyllomyces dendrorh...   288    3e-97
gb|KOO25945.1|  hypothetical protein Ctob_004479 [Chrysochromulin...  38.1    1.6  
ref|WP_038038123.1|  hypothetical protein [Thermorudis peleae]        38.1    1.8  
gb|AHI06269.1|  hypothetical protein BDW_08845 [Bdellovibrio bact...  37.0    3.8  
ref|WP_025308474.1|  hypothetical protein [Bdellovibrio bacteriov...  37.0    4.3  
ref|WP_008739560.1|  hypothetical protein [Alcanivorax pacificus]...  36.2    7.8  


 >emb|CED82143.1| hypothetical protein [Xanthophyllomyces dendrorhous]
Length=142

 Score =  288 bits (738),  Expect = 3e-97, Method: Compositional matrix adjust.
 Identities = 142/142 (100%), Positives = 142/142 (100%), Gaps = 0/142 (0%)

Query  1    MQRAQQTSRVISRSIRPSMSMACRWYSSLGSSAGSPVLSLSPETITKSPTVHHGWPGSLY  60
            MQRAQQTSRVISRSIRPSMSMACRWYSSLGSSAGSPVLSLSPETITKSPTVHHGWPGSLY
Sbjct  1    MQRAQQTSRVISRSIRPSMSMACRWYSSLGSSAGSPVLSLSPETITKSPTVHHGWPGSLY  60

Query  61   PSRKGLKKIAPRPSKEERKLQSLRSKEANKREAELDAFRRRNLQIFIEKFVSDSAKLPYP  120
            PSRKGLKKIAPRPSKEERKLQSLRSKEANKREAELDAFRRRNLQIFIEKFVSDSAKLPYP
Sbjct  61   PSRKGLKKIAPRPSKEERKLQSLRSKEANKREAELDAFRRRNLQIFIEKFVSDSAKLPYP  120

Query  121  KFDKILCPPYVPPGGRDGKRDL  142
            KFDKILCPPYVPPGGRDGKRDL
Sbjct  121  KFDKILCPPYVPPGGRDGKRDL  142


>gb|KOO25945.1| hypothetical protein Ctob_004479 [Chrysochromulina sp. CCMP291]
Length=455

 Score = 38.1 bits (87),  Expect = 1.6, Method: Composition-based stats.
 Identities = 28/84 (33%), Positives = 42/84 (50%), Gaps = 13/84 (15%)

Query  30   GSSAGSPVLSLSPETITKSPTVHHGWPGS---LYPSRKGLK-------KIAPRPSKEERK  79
            G SA   +++L P  +      HHGW G    L  +++GL+       K+ PRP ++ ++
Sbjct  80   GPSAIKMIVTLPPYLMLDGFAEHHGWDGVKAYLDLAKRGLRIELQLASKVLPRPKEQVKQ  139

Query  80   LQSLRSKEANKRE---AELDAFRR  100
            L SL   EA+K E    E D  RR
Sbjct  140  LGSLMVCEADKVERLRREFDYLRR  163


>ref|WP_038038123.1| hypothetical protein [Thermorudis peleae]
Length=295

 Score = 38.1 bits (87),  Expect = 1.8, Method: Compositional matrix adjust.
 Identities = 30/93 (32%), Positives = 42/93 (45%), Gaps = 19/93 (20%)

Query  30   GSSAGSPVLSLSPETITKSPTVHHGWPGSLYPSRKGLKKIAPRPSKEERKLQSLRSKEAN  89
            G++ GS  L L  + I     V  G P +L  S++             RKL +LR    N
Sbjct  149  GANGGSLTLDLMDQLI---DLVKPGRPDALLMSKR-----------SRRKLSALRRASGN  194

Query  90   KREAELDAFRRRNLQ-----IFIEKFVSDSAKL  117
              E ++DAF RR L      I ++ F+SDS  L
Sbjct  195  LLETDVDAFGRRALFYDGIPILVDDFISDSETL  227


>gb|AHI06269.1| hypothetical protein BDW_08845 [Bdellovibrio bacteriovorus W]
Length=448

 Score = 37.0 bits (84),  Expect = 3.8, Method: Composition-based stats.
 Identities = 25/83 (30%), Positives = 39/83 (47%), Gaps = 7/83 (8%)

Query  42   PETITKSPTVHHGWPGSLYPSRKGLKKIAPRPSKEERKLQSLRSKEANKREAELDAFRRR  101
            PE +T S +V  G   SLYP  + L K AP   K +  L   + KE  K E   D     
Sbjct  303  PEILTNSSSVFQGVEMSLYPLLEALTKDAPHKEKAQTILN--QCKELLKDEVTFD-----  355

Query  102  NLQIFIEKFVSDSAKLPYPKFDK  124
             ++ FI  ++++   L +  ++K
Sbjct  356  EIKTFINAYLTNPLTLEFTSYEK  378


>ref|WP_025308474.1| hypothetical protein [Bdellovibrio bacteriovorus]
Length=430

 Score = 37.0 bits (84),  Expect = 4.3, Method: Composition-based stats.
 Identities = 25/83 (30%), Positives = 39/83 (47%), Gaps = 7/83 (8%)

Query  42   PETITKSPTVHHGWPGSLYPSRKGLKKIAPRPSKEERKLQSLRSKEANKREAELDAFRRR  101
            PE +T S +V  G   SLYP  + L K AP   K +  L   + KE  K E   D     
Sbjct  285  PEILTNSSSVFQGVEMSLYPLLEALTKDAPHKEKAQTILN--QCKELLKDEVTFD-----  337

Query  102  NLQIFIEKFVSDSAKLPYPKFDK  124
             ++ FI  ++++   L +  ++K
Sbjct  338  EIKTFINAYLTNPLTLEFTSYEK  360


>ref|WP_008739560.1| hypothetical protein [Alcanivorax pacificus]
 gb|AJD47396.1| hypothetical protein S7S_04870 [Alcanivorax pacificus W11-5]
Length=303

 Score = 36.2 bits (82),  Expect = 7.8, Method: Compositional matrix adjust.
 Identities = 26/73 (36%), Positives = 41/73 (56%), Gaps = 5/73 (7%)

Query  3    RAQQTSRVISRSIRPSMSMACRWYSSLGSSA--GSPVLSLSPETITKSPTVHHGWPGSLY  60
            R QQ  R+  +  +P +SMAC   +SL  +   G+ ++ LSP  + ++PT H G PG  +
Sbjct  201  RQQQAMRLKQKPAQPWISMACHDQASLAHAVALGADMVLLSP--VRETPT-HPGQPGMGW  257

Query  61   PSRKGLKKIAPRP  73
            PS + L +  P P
Sbjct  258  PSFRALAEGLPLP  270


Lambda      K        H        a         alpha
   0.320    0.134    0.406    0.792     4.96 

Gapped
Lambda      K        H        a         alpha    sigma
   0.267   0.0410    0.140     1.90     42.6     43.6 

Effective search space used: 641414800915


  Database: nr
    Posted date:  Sep 23, 2015 12:05 AM
  Number of letters in database: 26,053,659,533
  Number of sequences in database:  71,551,133


Matrix: BLOSUM62
Gap Penalties: Existence: 11, Extension: 1
Neighboring words threshold: 11
Window for multiple hits: 40
```
